# Supplementary material for: Prevalence and genotype distribution of Human Papillomavirus (HPV) among 14,110 women in Anqing urban area: A population-based cross-sectional survey
Source: PLoS One. 2025 Dec 1;20(12):e0336959. doi: 10.1371/journal.pone.0336959 (PMC12668523; doi:10.1371/journal.pone.0336959)
Supplement: S1 Table — (PDF) [file pone.0336959.s001.pdf]

	Genotype	2022	2023	2024	total
Lr-HPV genotype	6	8(0.18)	11(1.23)	21(0.42)	40(0.28)
	11	7(0.16)	10(0.21)	4(0.08)	21(0.15)
	42	24(0.55)	22(0.46)	25(0.51)	71(0.50)
	43	20(0.46)	24(0.50)	22(0.45)	66(0.47)
	81	64(1.47)	63(1.30)	92(1.86)	219(1.55)
	83	3(0.07)	1(0.02)	1(0.02)	5(0.04)
Hr-HPV Genotype	16	59(1.36)	54(1.12)	57(1.15)	170(1.20)
	18	23(0.53)	26(0.54)	16(0.32)	65(0.46)
	31	9(0.21)	14(0.29)	19(0.38)	42(0.30)
	33	27(0.62)	26(0.54)	24(0.49)	77(0.55)
	35	3(0.07)	7(0.14)	8(0.16)	18(0.13)
	39	16(0.37)	16(0.33)	10(0.20)	42(0.30)
	45	3(0.07)	1(0.02)	9(0.18)	13(0.09)
	51	31(0.71)	33(0.68)	33(0.67)	97(0.69)
	52	110(2.53)	120(2.48)	162(3.28)	392(2.78)
	53	41(0.94)	62(1.28)	60(1.21)	163(1.16)
	56	20(0.46)	18(0.37)	17(0.34)	55(0.39)
	58	52(1.20)	61(1.26)	88(1.78)	201(1.42)
	59	16(0.37)	20(0.41)	16(0.32)	52(0.37)
	66	10(0.25)	10(0.21)	12(0.24)	32(0.23)
	68	26(0.60)	24(0.50)	26(0.53)	76(0.54)
	73	1(0.02)	3(0.06)	2(0.04)	6(0.04)
	82	4(0.09)	1(0.02)	6(0.12)	11(0.078)
Multiple infections	double infection	141(3.25)	167(3.45)	234(4.73)	542(3.84)
	triple infection	36(0.83)	35(0.72)	64(1.29)	135(0.96)
	quadruple infection	14(0.32)	16(0.33)	16(0.32)	46(0.32)
	quintuple infection	3(0.07)	8(0.17)	9(0.18)	20(0.14)
Total		771(17.79)	853(17.65)	1053(21.30)	2677(18.97)
