# Supplementary material for: Prevalence and genotype distribution of Human Papillomavirus (HPV) among 14,110 women in Anqing urban area: A population-based cross-sectional survey
Source: PLoS One. 2025 Dec 1;20(12):e0336959. doi: 10.1371/journal.pone.0336959 (PMC12668523; doi:10.1371/journal.pone.0336959)
Supplement: S2 Table — (PDF) [file pone.0336959.s002.pdf]

Genotype		<20 (n=48)	20~29 (n=806)	30~39 (n=2826)	40~49 (n=4590)	50~59 (n=4702)	>59 (n=1138)	P
Lr-HPV genotype	6*	3(6.25)	6(0.74)	16(0.57)	26(0.57)	23(0.49)	9(0.79)	0.002
	11*	7(14.58)	5(0.62)	9(0.32)	11(0.24)	18(0.38)	2(0.18)	<0.01
	42*	1(2.08)	18(2.23)	28(0.99)	410(8.93)	48(1.02)	9(0.79)	<0.01
	43*	1(2.08)	12(1.49)	27(0.96)	468(10.20)	60(1.28)	12(1.05)	<0.01
	81*	4(8.33)	18(2.23)	60(2.12)	124(2.70)	156(3.32)	42(3.69)	<0.01
	83	0(0)	0(0)	2(0.07)	5(0.11)	4(0.09)	2(0.18)	0.795
Hr-HPV genotype	16*	5(10.42)	23(2.85)	63(2.23)	79(1.72)	84(1.79)	26(2.28)	0.001
	18	1(2.08)	11(1.36)	21(0.74)	33(0.72)	54(1.15)	11(0.97)	0.149
	31*	0(0)	4(0.50)	224(7.93)	20(0.44)	24(0.51)	16(1.41)	<0.01
	35*	0(0)	4(0.50)	7(0.25)	14(0.31)	19(0.40)	12(1.05)	0.023
	39	0(0)	5(0.62)	22(0.78)	16(0.35)	30(0.64)	6(0.53)	0.204
	45	0(0)	1(0.12)	5(0.18)	7(0.15)	12(0.26)	5(0.44)	0.41
	51*	2(4.17)	19(2.36)	33(1.17)	63(1.37)	717(15.25)	18(1.58)	<0.01
	52*	3(6.25)	35(4.34)	118(4.18)	186(4.05)	836(17.78)	65(5.71)	<0.01
	53*	3(6.25)	15(1.86)	54(1.91)	85(1.85)	686(14.59)	37(3.25)	<0.01
	56*	2(4.17)	10(1.24)	29(1.03)	38(0.83)	420(8.93)	21(1.85)	<0.01
	58*	1(2.08)	23(2.85)	68(2.41)	94(2.05)	416(8.85)	44(3.87)	<0.01
	59*	4(8.33)	14(1.74)	22(0.78)	42(0.92)	293(6.23)	6(0.53)	<0.01
	66*	1(2.08)	10(1.24)	21(0.74)	10(0.22)	27(0.57)	59(5.18)	<0.01
	68*	0(0)	10(1.24)	32(1.13)	57(1.24)	52(1.11)	72(6.33)	<0.01
	73	0(0)	4(0.05)	6(0.21)	8(0.17)	4(0.09)	0(0)	0.111
	82*	0(0)	1(0.12)	4(0.14)	3(0.07)	8(0.17)	10(0.88)	0.004
total		38(79.17)	248(30.77)	871(30.82)	1799(39.19)	3991(84.88)	484(42.53)	
