# Supplementary material for: Prevalence and genotype distribution of Human Papillomavirus (HPV) among 14,110 women in Anqing urban area: A population-based cross-sectional survey
Source: PLoS One. 2025 Dec 1;20(12):e0336959. doi: 10.1371/journal.pone.0336959 (PMC12668523; doi:10.1371/journal.pone.0336959)
Supplement: S3 Table — (PDF) [file pone.0336959.s003.pdf]

Genotype		NILM (n=2302)	ASC-US (n=204)	ASC-H (n=78)	LSIL (n=49)	HSIL (n=44）
Lr-HPV genotype	6	72(3.13)	8(3.92)	2(2.56)	1(2.04)	0(0)
	11	38(1.65)	8(3.92)	2(2.56)	2(4.08)	2(4.55)
	42	130(5.65)	7(3.43)	2(2.56)	6(12.24)	1(2.27)
	43	149(6.47)	8(3.92)	1(1.28)	2(4.08)	0(0)
	81	364(15.81)	22(10.78)	8(10.26)	5(10.20)	0(0)
	83	10(0.43)	2(0.98)	1(1.28)	0(0)	0(0)
Hr-HPV Genotype	16	210(9.12)	29(14.22)	21(26.92)	3(6.12)	17(38.64)
	18	108(4.69)	13(6.37)	3(3.85)	3(6.12)	5(11.36)
	31	66(2.87)	8(3.92)	0(0)	2(4.08)	1(2.27)
	35	47(2.04)	5(2.45)	1(1.28)	1(2.04)	2(4.55)
	39	64(2.78)	8(3.92)	4(5.13)	3(6.12)	0(0)
	45	28(1.22)	0(0)	0(0)	0(0)	2(4.55)
	51	167(7.25)	14(6.86)	7(8.97)	9(18.37)	3(6.82)
	52	531(23.07)	61(29.90)	24(30.77)	10(20.41)	4(9.09)
	53	276(11.99)	31(15.20)	10(12.82)	5(10.20)	1(2.27)
	56	126(5.47)	10(4.90)	4(5.13)	5(10.20)	3(6.82)
	58	285(12.38)	29(14.22)	16(20.51)	10(20.41)	10(22.73)
	59	97(4.21)	6(2.94)	2(2.56)	3(6.12)	2(4.55)
	66	51(2.22)	11(5.39)	6(7.69)	5(10.20)	1(2.27)
	68	145(6.30)	12(5.88)	3(3.85)	2(4.08)	2(4.55)
	73	19(0.83)	1(0.49)	2(2.56)	0(0)	0(0)
	82	13(0.56)	1(0.49)	2(2.56)	1(2.04)	2(4.55)
