# Supplementary material for: Prevalence and genotype distribution of Human Papillomavirus (HPV) among 14,110 women in Anqing urban area: A population-based cross-sectional survey
Source: PLoS One. 2025 Dec 1;20(12):e0336959. doi: 10.1371/journal.pone.0336959 (PMC12668523; doi:10.1371/journal.pone.0336959)
Supplement: S4 Table — (PDF) [file pone.0336959.s004.pdf]

Types of Infection	total	NILM	ASC-US	ASC-H	LSIL	HSIL
single infections	1936	1708(88.22%)	124(7.26%)	46(2.38%)	26(1.34%)	32(1.65%)
Multiple infections	741	607(81.92%)	33(4.45%)	64(8.64%)	25(3.37%)	12(1.62%)
$\chi 2$		18.228	3.697	53.313	11.827	0.004
P		<0.001	0.055	<0.001	0.001	0.951
