# Supplementary material for: Prevalence and genotype distribution of Human Papillomavirus (HPV) among 14,110 women in Anqing urban area: A population-based cross-sectional survey
Source: PLoS One. 2025 Dec 1;20(12):e0336959. doi: 10.1371/journal.pone.0336959 (PMC12668523; doi:10.1371/journal.pone.0336959)
Supplement: Attachment — Submitted filename: Response to Reviewers.docx [file pone.0336959.s006.docx]

Dear Editor and Reviewers,

We sincerely thank you for handling our manuscript and for the constructive comments provided by the reviewers. We are particularly encouraged that both reviewers found the manuscript to be technically sound and agreed that the data support the conclusions

We have carefully considered all the suggestions and have revised the manuscript accordingly. We believe that these changes have significantly improved the quality of our work. Our point-by-point responses to the reviewers' comments are detailed below.

Response to Editor:

**Comment 1**: Please state what role the funders took in the study. If the funders had no role, please state: "The funders had no role in study design, data collection and analysis, decision to publish, or preparation of the manuscript."

Response: Thank you for this important comment. Our funders had no role in any aspect of this study. As suggested, we have now included the following statement in the 'Acknowledgments' section of the revised manuscript: "The funders had no role in study design, data collection and analysis, decision to publish, or preparation of the manuscript."

**Comment 2**: Please complete your Competing Interests on the online submission form to state any Competing Interests. If you have no competing interests, please state "The authors have declared that no competing interests exist."

Response: We have received your message concerning the Competing Interests declaration for our submission.This is to confirm that we have no competing interests. As per the journal's policy, we have already stated "The authors have declared that no competing interests exist." in the designated field of the online submission form.

**Comment 3**: PLOS requires an ORCID iD for the corresponding author in Editorial Manager on papers submitted after December 6th, 2016. Please ensure that you have an ORCID iD and that it is validated in Editorial

Response: Thank you for your suggestion regarding the ORCID iD. We have followed your advice and successfully registered for an ORCID. My ORCID iD is: <https://orcid.org/0000-0002-1999-061X>. I believe this is a valuable practice for academic identity management, and I appreciate you bringing it to my attention.

**Comment 4**: Please include captions for your Supporting Information files at the end of your manuscript, and update any in-text citations to match accordingly. Please see our Supporting Information guidelines for more information: <http://journals.plos.org/plosone/s/supporting-information>.

If the reviewer comments include a recommendation to cite specific previously published works, please review and evaluate these publications to determine whether they are relevant and should be cited. There is no requirement to cite these works unless the editor has indicated otherwise.

Please review your reference list to ensure that it is complete and correct. If you have cited papers that have been retracted, please include the rationale for doing so in the manuscript text, or remove these references and replace them with relevant current references. Any changes to the reference list should be mentioned in the rebuttal letter that accompanies your revised manuscript. If you need to cite a retracted article, indicate the article’s retracted status in the References list and also include a citation and full reference for the retraction notice.

Response: Thank you for your comments on our manuscript titled "Prevalence and genotype distribution of Human Papillomavirus (HPV) among 14,110 women in Anqing urban area: a population-based cross-sectional survey". We have carefully revised the manuscript according to the editorial request regarding the Supporting Information. The specific changes we have made are as follows:

We have added a "Supporting Information" section at the end of the manuscript file, immediately following the Reference list. In this section, we have provided descriptive captions for all Supporting Information files.

We have updated all in-text citations to the Supporting Information throughout the manuscript to ensure they are formatted correctly and match the captions accordingly (e.g.,(S1 Fig), (S1 Table)).

These changes have been incorporated into the revised manuscript file we have submitted.

We believe that the manuscript now fully complies with the journal's guidelines for Supporting Information. We appreciate the time and effort you and the reviewers have dedicated to our work.

**Comment 5**: Has the statistical analysis been performed appropriately and rigorously?

Reviewer #1: I Don't Know

Reviewer #2: No

Dear Reviewer #1 and #2:

Thank you for raising this important question regarding the statistical analysis. We agree that rigorous statistics are fundamental to drawing reliable conclusions. We confirm that the statistical analysis in our manuscript has been performed appropriately and rigorously. The choice of statistical tests was determined by the experimental design and the nature of the data.

For comparisons among aged- specific distribution of HPV infections groups, we used Fisher’s chi-square test, as appropriate. Because the infection rates of HPV genotypes were statistically low when grouped by age, we chose the more accurate Fisher's exact test to compare the positive rates across different groups. The Chi-square test was used to compare the effects of single infections and multiple infections across different cytological grades. A dedicated "Statistical Analysis" subsection in the Materials and Methods details all tests used, the software (SPSS 22.0), and the significance threshold (α = 0.05). We have defined statistical significance P < 0.05, and all specific p-values were reported.

In summary, we have taken great care to ensure that our statistical approach is robust, appropriate for our data, and transparently reported. We are confident that the conclusions drawn are firmly supported by the statistical evidence.

We thank the reviewer for their thorough review and hope this clarification fully addresses their concern.

**Comment 6**: Add a graphical scheme of the findings besides the tables to ease the reading

Response: Thank you for the suggestion. A new figure (**Fig. 1**) presenting the distribution of HPV genotypes from 2022 to 2024 has been inserted into the manuscript.

**Comment 7**: Comparison of single and multiple HPV infections across cervical lesions with Statistical Assessment

Response: A new table (**Table 4**) was added to comparing single vs. multiple infections across cytological grades with statistical analysis.

**Comment 8**: Added discussion of COVID-19 pandemic impact and potential HPV genotype shifts.

Response: As recommended, we have added this content to the third paragraphs of the Introduction and the fourth paragraphs of Discussion. All changes have been highlighted in yellow.

**Comment 9:** Comparison with the regions from Asia could be valuable to draw the differences or similarities, considering oral HPV infection as a trend in global population and suggestion for further investigation in Chinese population.

Response: We have revised the manuscript to include the suggested content in the designated sections: analysis of HPV genotypes in Asia has been added to the second paragraph of the Discussion and a context of oral HPV infection has been added to the eighth paragraph. These additions are also marked in yellow.

**Comment 10:** Correct LISL and HISL to LSIL and HSIL. In my opinion, the percentage is incorrect or the group description is incorrect.

Response: We sincerely thank the reviewer for this critical observation and for highlighting these important issues. We have thoroughly reviewed the entire manuscript and corrected all instances of "LISL" to "LSIL" and "HISL" to "HSIL".

**Comment 11**: The overall prevalence of HPV infection in the study population was 18.97%, while all subgroup rates exceed 42%. Please explain this discrepancy.

Response: We thank the reviewer for this critical observation. The percentages in the abstract and this section represent the HPV positivity rate within each age group (positive cases/total tested in that age group), not the percentage among all women tested. This explains why percentages exceed the overall 18.97% prevalence.

**Comment 12**: Expanded introduction section to include discussion of HPV genotyping, phenotyping, and COVID-19 pandemic impact.

Response: As recommended, we have added this content to the fourth paragraphs of the Introduction. All changes have been highlighted in yellow.

**Comment 13**: There is no information whether the patients were vaccinated or not. Vaccination affects the recurrence of the disease and persistent infections.

Response: We apologize for the lack of data on HPV vaccination status in our hospital's information system; therefore, this variable could not be included in the analysis.

**Comment 14:** I suggest writing LSIL- CIN 1 and HSIL CIN 2 and CIN 3 according to the LATS terminology, which is clearer for histopathological diagnoses.

Response: We thank the reviewer for the valuable suggestion regarding the classification of cervical lesions. While we acknowledge that histopathological grading offers high specificity, the current analysis is primarily based on cytological findings (TCT), as this constitutes the most routinely available and standardized screening data in our clinical setting. To ensure internal consistency across all our data, we have maintained the cytological classification (e.g., ASC-US, LSIL, HSIL) for this study.

**Comment 15:** Added comparison with European data.

Response: As recommended, we have added this content to the second paragraphs of the Discussion. All changes have been highlighted in yellow.
